# Endoscopic Resection of a Rare Inverted Papilloma Involving the Lacrimal Duct and Sac

**DOI:** 10.7759/cureus.107252

**Published:** 2026-04-17

**Authors:** Camila S Ríos de Choudens, Antonio Bures Rodriguez, Ana Melero Pardo, Francisco Del Valle, Juan C Portela Arraiza

**Affiliations:** 1 Department of Otolaryngology, Head and Neck Surgery, University of Puerto Rico, Medical Sciences Campus, San Juan, PRI

**Keywords:** endoscopic dacryocystorhinostomy, endoscopic surgery, inverted papilloma, lacrimal duct, lacrimal sac

## Abstract

Inverted papilloma (IP) is a benign epithelial tumor. Most cases occur unilaterally in males in their 60s to 70s. Extension into the lacrimal duct or sac is uncommon and presents diagnostic challenges due to overlapping symptoms with more common conditions, such as dacryocystitis. Their nonspecific presentation may lead to delayed or incorrect diagnosis. Preoperative imaging, such as CT or MRI, is essential for determining tumor extent. Complete surgical excision, often achieved through endoscopic approaches, is the gold standard for treatment and minimizes the risk of recurrence.

We present the case of a 58-year-old male with a 5-year history of a progressively enlarging medial left lower eyelid mass, epiphora, and occasional bloody discharge. The patient had a history of nasal polyposis, with two prior surgeries for nasal polyp removal. Examination revealed a medial canthal mass without infection. Our team performed a biopsy at the outpatient clinic, and histopathology confirmed the diagnosis of IP. The patient underwent a left dacryocystorhinostomy with tumor resection. We completed the procedure without complications.

This case highlights the importance of considering IP in the differential diagnosis of medial canthal masses with epiphora. Prompt recognition and effective surgical management are crucial for preventing recurrence and optimizing outcomes.

## Introduction

Inverted papilloma (IP) is a rare benign epithelial tumor that primarily affects the nasal cavity and paranasal sinuses. IPs make up about 0.5% to 4% of all nasal tumors, and around 70% of all sinonasal papillomas [1]. IPs typically occur unilaterally and are more common in males in their 60s and 70s.

IPs exhibit a unique growth pattern, in which epithelial cells grow inward into the underlying stroma, often resulting in significant local destruction and potential malignant transformation. IPs arise from the mucosal epithelial lining of the sinonasal tract, which connects to the nasolacrimal duct and lacrimal sac, with most cases occurring in the nasal cavity and paranasal sinuses [1,2].

The involvement of the lacrimal duct or sac by IPs is an infrequent occurrence, presenting diagnostic and therapeutic challenges due to the potential for nasolacrimal duct obstruction, epiphora, and visual disturbance. The most common symptoms experienced by IPs are unilateral nasal obstruction, rhinorrhea or purulent discharge, and epistaxis. However, once this lesion extends into the nasolacrimal system, ocular symptoms may occur, such as epiphora, ocular discharge, and a medial canthal lesion. Extension to these structures is often overlooked, as symptoms are frequently attributed to more common causes, such as dacryocystitis or nasolacrimal duct obstruction. Given the rarity of this phenomenon, clinicians must consider IPs in the differential diagnosis when encountering patients with unilateral visual disturbances or epiphora with no ophthalmologic causes. This case report presents a rare instance of an IP with extension into the lacrimal duct and sac.

## Case presentation

We present the case of a 58-year-old male with a 5-year history of a slowly enlarging, medial left lower eyelid mass. Upon initial evaluation by the Oculoplastic team, the patient described a left eyelid mass that gradually increased in size, with associated epiphora and occasional bloody secretions from the left eye. He denied any signs of infection, erythema, prior ocular or orbital pathologies, and history of trauma or systemic illness. However, it was noted that the patient's past medical history was notable for nasal polyposis with two prior sinonasal surgeries. The report by the Oculoplastics team mentions a left lower eyelid mass that was associated with the medial canthus. Given the persistence and gradual enlargement of the lesion, an Oculoplastic surgeon performed an excision of the lesion. The pathology report confirmed the diagnosis of an IP.

**Figure 1:**
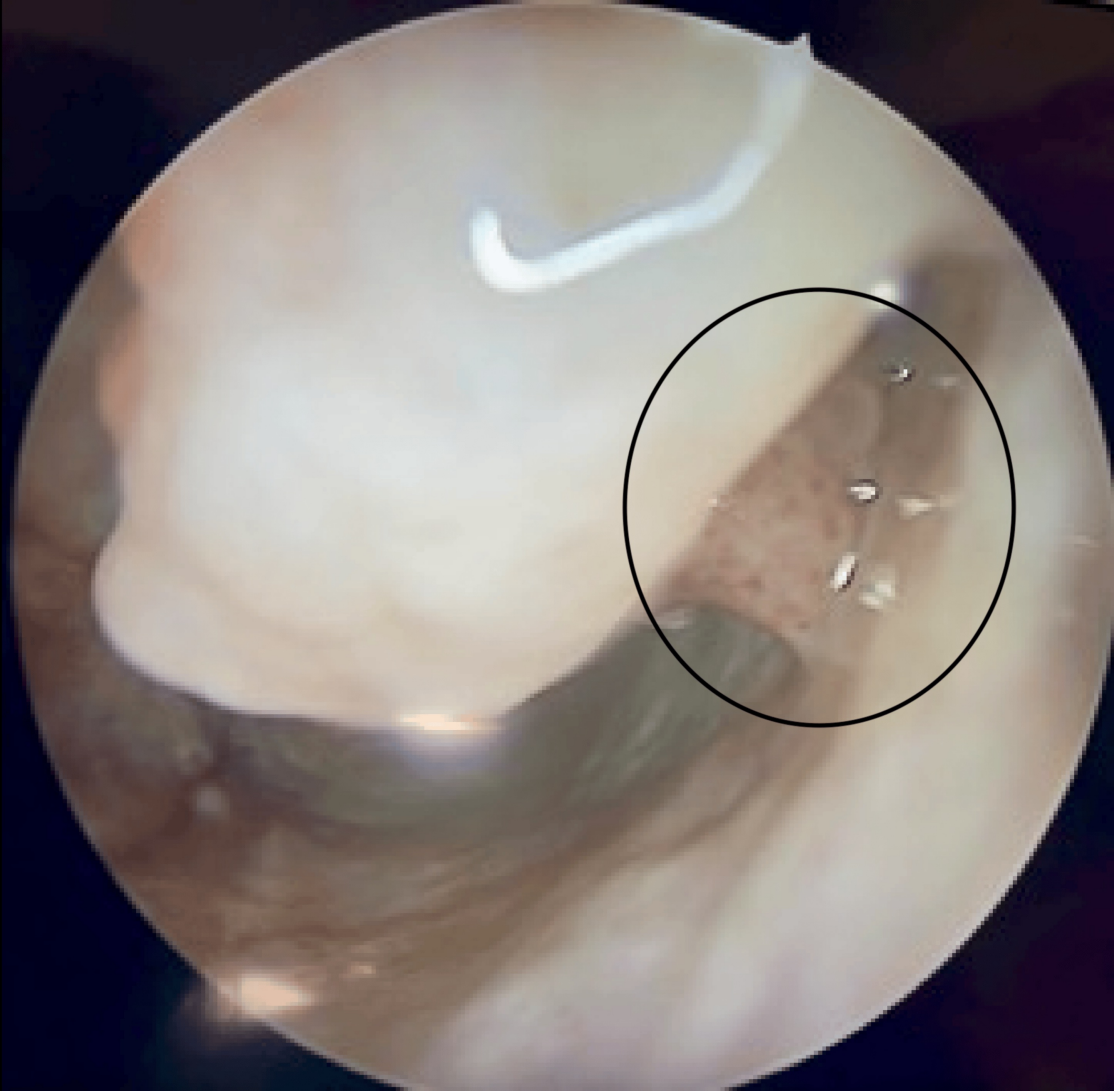
Nasal endoscopy revealed a mass in the left inferior meatus exhibiting characteristic features of inverted papilloma.

**Figure 2:**
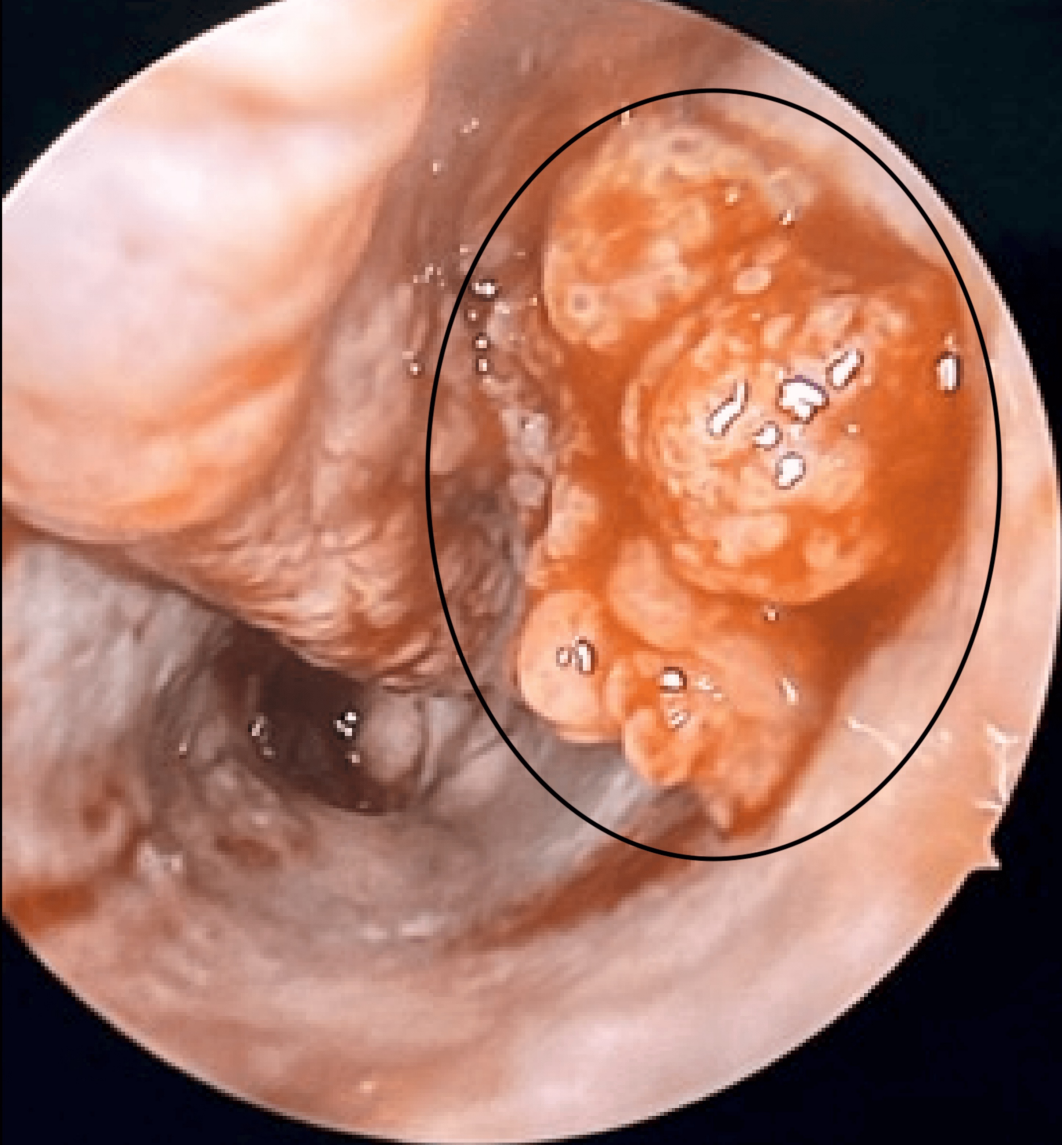
Nasal endoscopy following fracture of the inferior turbinate reveals an inverted papilloma in the inferior meatus.

**Figure 3:**
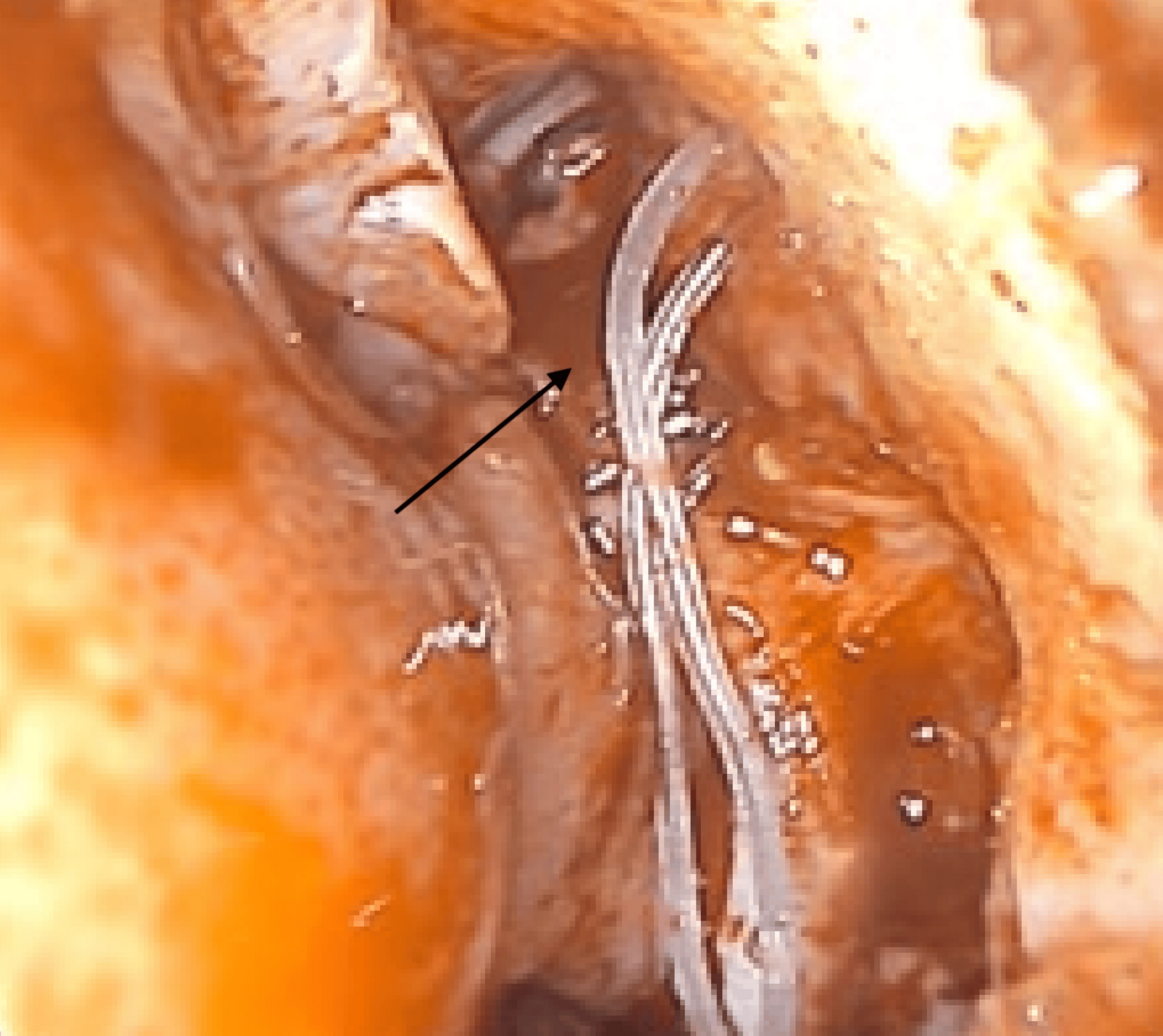
Crawford stents were inserted through the puncta and retrieved via the nasal cavity following the removal of the inverted papilloma.

Subsequently, the Oculoplastic Service referred the patient to our Otolaryngology clinic. Nasal endoscopy revealed a mass in the left inferior meatus, suggesting potential sinonasal involvement in the development of the IP (Figures 1, 2). The patient underwent a left dacryocystorhinostomy with tumor resection. A mucosal flap was elevated anterior to the uncinate process and superior to the inferior turbinate, then elevated posteriorly and divided horizontally for protection. Using a high-speed 4 mm diamond burr and Kerrison rongeurs, the bone overlying the nasolacrimal duct and sac was removed. The left upper and lower puncta were dilated with a punctal dilator, and a Bowman probe was passed through the canaliculus to the nasal bony stop. A superiorly based mucosal flap was elevated to encompass the tumor and was excised. Crawford stents were placed through the puncta and retrieved in the nasal cavity, secured with a Prolene suture at the left vestibule (Figure 3). The mucosal flaps were re-draped, gel foam was applied, and the IT stump was cauterized. The patient tolerated the procedure without complications. Pathology revealed no adverse features, with no evidence of focal dysplasia. The patient has followed up at clinic appointments with no evidence of recurrence for 12 months since the surgical excision.

## Discussion

The lacrimal drainage system is a rare primary site for IP, with only a limited number of cases reported in the literature. Papillomas in the lacrimal system typically present with symptoms such as epiphora and a mass near the medial canthus if diagnosed early. These symptoms are consistent with the case presented. In more advanced stages, they may lead to proptosis, skin ulceration, and invasion into surrounding structures [1]. Preoperative imaging is crucial for assessing the extent of the lesion and its potential invasion into nearby structures. Both CT and MRI are considered complementary modalities for pretreatment staging of IP, as they provide accurate information on the spread of the disease and help distinguish the tumor from inflamed mucosa, retained secretions, or granulation tissue [3].

Over the past 25 years, the approach to treating sinonasal IP has evolved, particularly with advancements in endoscopic surgery. Traditionally, medial maxillectomies via lateral rhinotomy or mid-facial degloving were considered the gold standard [2]. More recently, endoscopic and combined endoscopic-assisted procedures have shown comparable or even better recurrence rates than these more invasive techniques. When comparing these two methods, the endoscopic approach avoids any facial incisions and provides excellent visualization. A review by Lund et al. found a 14.5% recurrence rate at a 37-month follow-up in patients treated with pure endoscopic techniques, which was similar to the 16.7% recurrence rate at 62 months in those treated with lateral rhinotomy and medial maxillectomy [1]. Among patients undergoing limited resections, the recurrence rate was higher at 34.4% [1]. The location of the tumor plays a critical role in determining the best approach, and some authors recommend an endoscopic method, supplemented by extra-nasal techniques for tumors that are inaccessible by endoscopy, as observed in the case presented [4,5]. This combined approach aids in reducing the risk of recurrence. A meta-analysis indicated that piecemeal resection of IP via an endoscopic approach does not compromise tumor control compared with en bloc external techniques. The authors emphasized the importance of identifying and completely removing the tumor's attachment site [5]. In this case, the patient underwent endoscopic resection with stripping of the mucosa and bone drilling to ensure negative margins and complete removal of the attachment site. 

## Conclusions

This case report highlights the rare occurrence of an IP with extension into the lacrimal duct and sac, underscoring the diagnostic and therapeutic challenges associated with this pathology. Early recognition and a multidisciplinary approach, including expertise in otolaryngology and oculoplastic surgery, are paramount for optimal management. Advanced imaging techniques and meticulous surgical planning are crucial to accurately assess the extent of disease and achieve complete tumor excision while preserving critical structures. The patient's successful outcome underscores the importance of combining endoscopic and adjunctive surgical techniques, tailored to the tumor's location and extent. Continued long-term follow-up is essential to monitor for recurrence and ensure the durability of treatment outcomes. This case emphasizes the need for heightened clinical suspicion of IP in patients presenting with unexplained lacrimal system pathologies to prevent delayed diagnosis and associated complications.
